# Effect of Pretreatment of Activated Carbon on Iron Oxide-Loaded Catalysts to Significantly Enhance Production of Sebacic Acid from Castor Oil

**DOI:** 10.3390/molecules29184504

**Published:** 2024-09-23

**Authors:** Qingyun Zhang, Zhulin Wang, Zhichao Qin, Binglin Li, Zisheng Guo

**Affiliations:** 1 College of Food Science and Engineering, Northwest University, Xi’an 710069, China; 2 College of Food Science and Technology, Nanjing Agricultural University, Nanjing 210095, China; 3 Key Laboratory of Resources Biology and Biotechnology in Western China, Ministry of Education, College of Life Sciences, Northwest University, Xi’an 710069, China

**Keywords:** sebacic acid, Fe_2_O_3_, activated carbon, pretreatment, high yield

## Abstract

This study explores the efficient conversion of castor oil to sebacic acid utilizing iron oxide (Fe_2_O_3_) loaded on activated carbons as catalysts. Through a combination of saponification, acidification, and catalytic cracking, sebacic acid was produced with a notable yield improvement. The process involved using liquid paraffin as a thinning agent, overcoming the limitations of traditional toxic agents. The catalysts were prepared via adsorption-precipitation-calcination methods, with ultrasonication pretreatment to enhance iron adsorption on activated carbons. The chemical composition, structure, and morphology properties were investigated by different characterizations; such as scanning electron microscopy (SEM), thermogravimetric analysis (TG/DTG). Systematic investigations into the adsorption capacity, catalytic activity, and operational parameters like temperature, reaction time, and catalyst recycling were conducted. The optimized method achieved a sebacic acid yield of 83.4%, significantly higher than traditional methods (60.2%), with improved safety and environmental impact. The study provides valuable insights into sustainable and efficient sebacic acid production which is crucial for industrial applications in processing of castor oil.

## 1. Introduction

Castor oil, derived from the seeds of the *Ricinus communis* plant, is a versatile vegetable oil known for its high ricinoleic acid content. This unique composition has led to its extensive uses in both medicinal and industrial applications [1,2,3,4]. One prominent method of processing castor oil is alkali fusion, which transforms it into various chemical intermediates containing 8–10 carbon atoms [5]. Among them, sebacic acid is the most important product derived from alkali fusion. Sebacic acid, also known as decanedioic acid, is a dicarboxylic acid containing ten carbon atoms with the chemical formula C_10_H_18_O_4_ [5]. Sebacic acid is primarily used as a precursor in the production of polymers, notably nylon-610. Nylon-610 finds applications in a wide range of products, including textiles, automotive parts, and packaging materials [6,7,8,9]. Beyond polymers, sebacic acid is utilized to produce lubricants, plasticizers, and corrosion inhibitors. It also serves as a key ingredient in the synthesis of fragrances, dyes, and pharmaceuticals [1,5]

China plays a significant role in the global production of sebacic acid, with prominent production facilities such as Hebei Kaide Biomaterials Co., Ltd. (Hengshui, China) and Hebei Hengshui Jinghua Chemical Co., Ltd. (Hengshui, China). These factories boast annual production capacities exceeding 40,000 tons [10]. The production process in these facilities involves the alkali fusion of castor oil in a tank reactor, under high temperature (about 280 °C) and alkaline conditions (caustic pyrolysis) with phenol as a thinning agent. Despite these efforts, the yield typically remains below 65%, and the method raises environmental concerns and presents separation challenges. Meanwhile, this method poses environmental concerns and presents challenges in terms of separation. While various novel approaches for sebacic acid synthesis have been designed, such as microbial fermentation [11], microwave-induced [12], dihydroxylation [13], and electrolytic methods [14,15] using adipic acid, these techniques pose challenges for large-scale industrial applications. Consequently, enhancing the efficiency of the cracking process holds significant importance.

In this work, castor oil was converted into fatty acids firstly by saponification and acidification [16]. Then, they were catalyzed by NaOH and different Fe_2_O_3_ catalysts with the liquid paraffin as a thinning agent for the splitting decomposition. Finally, sebacic acid would be purified and recovered after the acidification and decolorization. Compared with reported toxic thinning agents, the production safety was improved significantly. Fe_2_O_3_ was attached on commercially activated carbons by the adsorption-precipitation-calcination method. The special attention was paid to the ultrasonication pretreatment of activated carbons in acidic oxidation solutions to improve its adsorption ability for iron. The chemical composition, structure, and morphology properties were investigated by different characterizations, such as scanning electron microscopy (SEM), and thermogravimetric analysis (TG/DTG). Sebacic acid was prepared with a satisfactory yield of 83.4% under the optimal condition, which was significantly increased than the traditional method. After being used five times, only the slight decrease was observed in the catalytic performances of Fe_2_O_3_. These unique physicochemical properties make this method a promising candidate for the industrial production of sebacic acid.

## 2. Results and Discussion

### 2.1. Variables Affecting Adsorption Capacity of Fe^3+^

**Figure 1:**
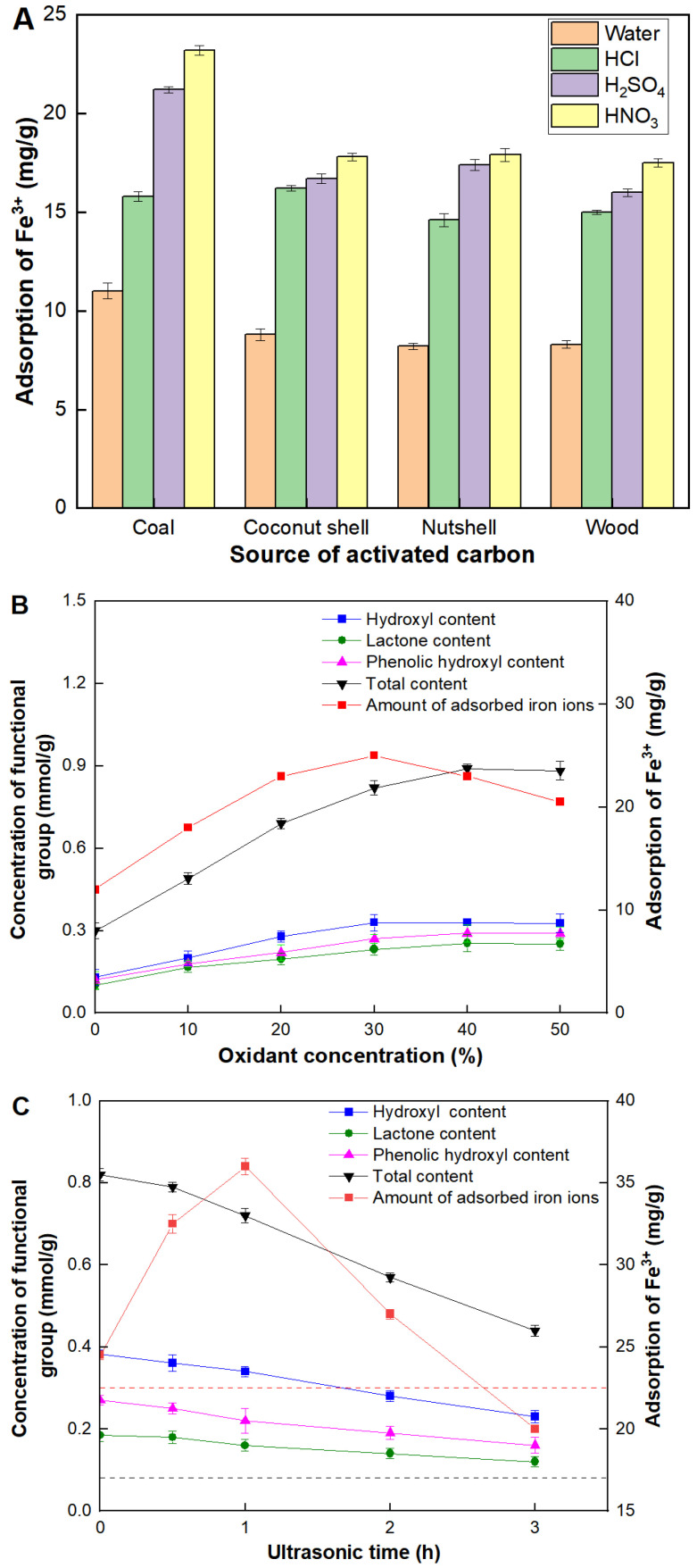
Variables affecting adsorption capacity of Fe^3+^. (**A**) Adsorption capacities of four kinds of commercial activated carbons. All oxidizing agent concentrations were set at 10%. (**B**) Effect of the concentration of the nitric acid; (**C**) Effect of the ultrasonic time. The red dashed line represented the adsorption capacity of CAC with ultrasonic treatment in the water. The black dash line represented the total content of functional groups of CAC with ultrasonic treatment in the water.

Four kinds of commercially activated carbons derived from coal, coconut shell, nutshell and wood, were used to screen their adsorption capacities for Fe^3+^. As shown in Figure 1A, the significant difference was observed in the adsorption capacity of Fe^3+^ under different elution processes. Compared with the deionized water, activated carbons eluted by acidic solutions showed an obvious increase in the adsorption capacity of Fe^3+^ in all cases. Among the acidic solutions, the elution efficiency from highest to lowest was nitric acid, sulfuric acid, and hydrochloric acid. This trend was attributed to oxidizing abilities of elution solutions. Acidic solutions enhanced the removal of impurities from the activated carbon surface, increasing the available sites for Fe^3+^ adsorption. Nitric acid, being a strong oxidizing agent, was the most effective, likely due to its ability to create more surface functional groups that favored Fe^3+^ binding [17]. After the treatment of low-oxidizing detergent solutions (deionized water and hydrochloric acid), there was no significant difference observed among the four kinds of commercially activated carbons. The minimal effect of deionized water and hydrochloric acid suggested that these solutions did not significantly modify the surface properties of the activated carbons. However, when high-oxidizing detergent solutions (nitric and sulfuric acid) were used, the Fe^3+^ adsorption capacity of coal-derived activated carbon (CAC) increased by approximately 27% compared to the other three types. This significant improvement highlighted the role of the oxidizing agent in enhancing the adsorption capacity of activated carbon. The specific structure and surface chemistry of CAC may have interacted more favorably with oxidizing agents, resulting in more efficient adsorption sites for Fe^3+^. Therefore, CAC was confirmed as the best candidate of activated carbons and nitric acid was used for the elution process.

**Figure 2:**
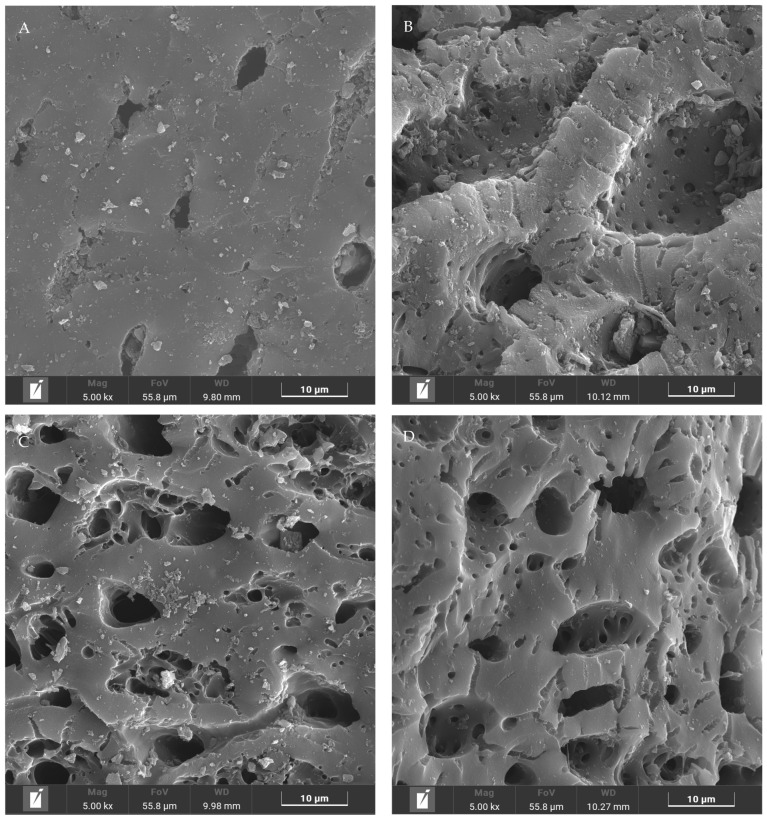
SEM analysis of CAC with different pretreatment. (**A**) initial state; (**B**) acid eluting; (**C**) ultrasonic treatment, and (**D**) acid eluting under the ultrasonic treatment.

Next, the concentration of nitric acid was investigated systematically, as shown in Figure 2B. It was observed that for nitric acid concentrations below 30%, the contents of carboxyl, hydroxyl, and lactone groups on the CAC surface increased with the nitric acid concentration. Correspondingly, the adsorption capacity of Fe^3+^ also increased. This trend can be attributed to the enhanced surface oxidation, which introduced more functional groups that serve as active sites for Fe^3+^ adsorption. However, for nitric acid concentrations above 30%, a decrease in adsorption capacity was observed, even though the maximum contents of carboxyl, hydroxyl, and lactone groups had not yet been reached. There were only slight increases in the contents of these functional groups at higher nitric acid concentrations. While the presence of carboxyl, hydroxyl, and lactone groups can potentially increase the number of active adsorption sites, leading to improved adsorption capacity, excessively high nitric acid concentrations led to detrimental effects on the CAC structure. Under extreme oxidizing conditions, such as those provided by high concentrations of nitric acid, the structure of CAC can be significantly compromised. The aggressive oxidative environment can cause pore collapse, reducing the surface area and pore volume, which are critical for effective adsorption. This structural degradation negates the benefits of the increased functional groups, resulting in a net decrease in adsorption capacity for Fe^3+^.

Special attention was paid on the ultrasonic treatment during the elution process. As shown in Figure 1C, the contents of functional groups and adsorption capacity of Fe^3+^ of CAC barely changed after the ultrasonic treatment in the deionized water. Although the ultrasonic treatment was slightly harmful to the increase in the contents of functional groups, a slight improvement was observed in the adsorption capacity of Fe^3+^. It might be because the ultrasonic treatment was beneficial for clearing pore channels. This phenomenon indicated that the content of functional groups might be beneficial for the adsorption of Fe^3+^, but they were not necessarily consistent, and the latter was decided by various factors. When 30% nitric acid was employed as the elution solution during the ultrasonic treatment, although the ultrasonic treatment was slightly harmful to the increase in contents of functional groups, a sharp change was observed in the adsorption capacity of Fe^3+^ with the ultrasound time. The maximum value of the adsorption capacity of Fe^3+^ reached 37.5 mg/g, which was 1.6 and 2.2 times higher than treatments of only nitric acid or ultrasound. Moreover, an additional experiment was carried out in which 30% nitric acid and ultrasound were respectively used to treat CAC. However, the adsorption capacity of Fe^3+^ only reached 30.7 mg/g, which was 1.2 times lower than our combination method. It might be explained that the ultrasonic treatment could clear the pore channel of CAC which should be beneficial for the interaction between nitric acid and the internal surface of CAC [18]. Meanwhile, the ultrasonic treatment might be harmful to the structure stability of the micropore or partial mesoporous of CAC particularly in the very long processing time.

**Figure 3:**
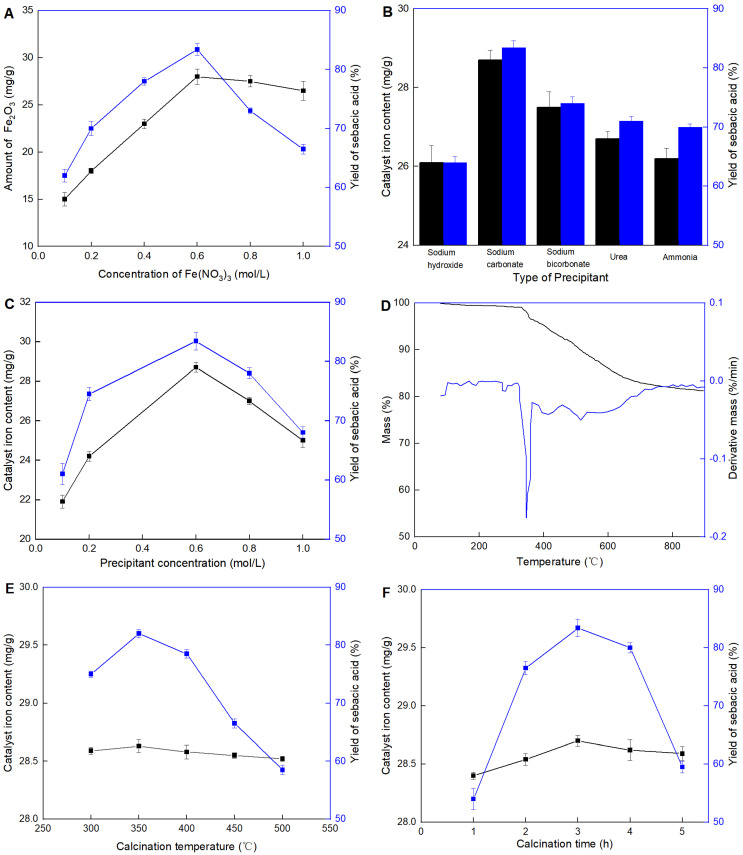
Preparation of Fe_2_O_3_/CAC. Effects of (**A**) concentration of Fe(NO_3_)_3_, (**B**) type of precipitant, (**C**) precipitant concentration, (**D**) TG/DTG curve, (**E**) calcination temperature, and (**F**) calcination time. All experiments belonged to the single-factor optimization experiment. Except the variable, other parameters were set as optimal conditions in each figure. The optimal conditions were as follows: 0.6 mol/L of concentration of Fe(NO_3_)_3_, 0.6 mol/L of Na_2_CO_3_ (precipitant), and 350 °C for 3 h in the calcination process.

The SEM analysis was used to study the effect of pretreatment on the structure and morphology of CAC. As shown in Figure 2A, the surface of the initial CAC was not smooth and flat, where the edge of the pore channel was clear, and its size ranged from 3–10 μm. After acid eluting, the outer surface of CAC was seriously destroyed, as shown in Figure 2B. Many internal channels were exposed leading to lower diffusional resistance for Fe^3+^. In fact, the ultrasonic treatment would also destroy the outer surface of CAC, as shown in Figure 2C. The size of the channel entrance was increased by over 20 μm. However, a lot of fragments were observed in this sample and some of them even blocked the channel entrance, which might be not a good phenomenon. Figure 3D shows the morphology of CAC with the pretreatment of acid eluting under the ultrasonic treatment. Although the outer surface of CAC was corroded, the pore channel became very smooth and flat, and the size of the channel entrance was increased by over 20 μm. Meanwhile, no fragment was observed on the channel entrance. More importantly, a lot of small pores were observed. During the pretreatment, they might be generated or were originally internal channels, but now, the outer surface was slightly corroded, causing them to be exposed. Although nitric acid was also used in Figure 2B, its distribution might be uneven, leading to more interaction with the outer surface. This phenomenon lead to a lot of channels being destroyed. In Figure 2D, nitric acid might diffuse into the pore channels well under the assistance of the ultrasonic treatment. Therefore, outer and internal channels could be modified simultaneously leading to the stronger adsorption ability for Fe^3+^.

### 2.2. Preparation of Fe_2_O_3_/CAC

The iron ion was adsorbed on the surface of pretreated CAC by impregnation and precipitation methods, which was further converted into Fe_2_O_3_ under calcination. Variables affecting catalytic performance were systematically investigated including Fe_2_O_3_ loading, precipitants and calcination processes.

The effect of the concentration of Fe(NO_3_)_3_ on the catalyst is shown in Figure 3A. It was observed that for the concentration of Fe(NO_3_)_3_ below 0.6 mol/L, the catalyst Fe_2_O_3_/CAC increased with the amount of Fe_2_O_3_, while for volumes above this value, a decrease in the activity was observed indicating that maximum activity was not necessarily consistent with the content of Fe_2_O_3_. Although Fe_2_O_3_ was the active site, it did not mean all Fe_2_O_3_ attached to the surface of CAC could participate in the reaction. Although Fe_2_O_3_ served as the active site, not all Fe_2_O_3_ attached to the surface of CAC participated in the reaction. At high loadings, Fe_2_O_3_ likely agglomerated randomly (similar to multi-layer adsorption or even aggregation), leading to mass transfer limitations for the substrate molecules trying to reach the active sites. This aggregation could block the diffusional channels of the CAC, reducing the overall effectiveness of the catalyst.

Precipitation was a crucial process during the preparation of Fe_2_O_3_/CAC. It not only directly determined the most amount of the iron attachment, but also affected the formation of Fe_2_O_3_ on the carrier surface. Five kinds of precipitants were evaluated, as shown in Figure 3B. Generally, precipitants with weak bases showed better performance in facilitating the attachment of iron on the carrier surface. When a strong base like NaOH was used, the precipitation formed rapidly, observable even to a naked eye. This phenomenon was actually caused by the aggregation of Fe(OH)_3_ rather than its effective attachment to the carriers. Consequently, very low catalytic performance was detected in the NaOH sample. Although NaHCO_3_ was the weakest base used in this work, the maximum catalytic performance was found in the Na_2_CO_3_ sample. This might be because if the base of the precipitant was too weak, it would affect the amount of iron precipitated. The findings suggest that the choice of precipitant played a significant role in the preparation of the Fe_2_O_3_/CAC catalyst. The weak bases appeared to provide more controlled and effective precipitation process, leading to better dispersion of Fe_2_O_3_ on the carrier surface. In contrast, strong bases caused rapid precipitation and aggregation, which negatively impacted the catalytic performance. The impurities introduced by urea or ammonia solutions could interfere with the catalytic reaction, possibly by blocking active sites or altering the surface properties of the catalyst.

After Na_2_CO_3_ was confirmed as the most suitable precipitant, the amount required was investigated systematically (Figure 3C). It was observed that the concentration of Na_2_CO_3_ at 0.5 mol/L and the catalytic performance both reached the maximum value. The concentration of Na_2_CO_3_ played a crucial role in determining the efficiency of the Fe_2_O_3_/CAC catalyst. At optimal concentrations, Na_2_CO_3_ facilitated the uniform precipitation of iron, ensuring that Fe_2_O_3_ was well-distributed across the carrier surface. This uniform distribution is critical for maximizing the available active sites and enhancing the catalytic performance. However, at lower concentrations of Na_2_CO_3_, insufficient precipitation resulted in poor iron loading. This deficiency meant fewer active sites were available for catalysis, leading to suboptimal catalytic performance. On the other hand, at higher Na_2_CO_3_ concentrations, the rapid and excessive precipitation likely caused iron ions to cluster together rather than disperse evenly. These clusters would create areas of high local concentration, which hindered the access of substrate molecules to the active sites due to mass transfer limitations.

Calcination was a crucial process for converting Fe(OH)_3_ into Fe_2_O_3_, ensuring the latter was firmly attached to the carrier surface. TG/DTG analysis was used to study the calcination process and confirm the optimal calcination conditions, as shown in Figure 3D. At temperatures between 300 and 500 °C, the decomposition of Fe(OH)_3_ to Fe_2_O_3_ was efficient, which was essential for creating a stable and active catalyst. The maximum mass loss rate was observed at 350 °C. When the heating temperature exceeded 500 °C, only slight mass loss was still observed.

Therefore, Fe_2_O_3_/CAC was prepared under different calcination temperatures from 300 to 500 °C to evaluate their catalytic activities during the production of sebacic acid. As shown in Figure 3E, the calcination temperature barely affected the iron loading but significantly influenced the catalytic activity of the final catalyst. When the temperature was too low and couldn’t reach the decomposition temperature of the precursor, the formation of the active component Fe_2_O_3_ on the carrier surface was reduced. Conversely, if the heating was too high, the decomposition occurred too rapidly, leading to the agglomeration of Fe_2_O_3_. This agglomeration affected its dispersion on the carrier surface, thereby reducing the catalytic activity. This finding highlighted the importance of optimizing the calcination temperature to ensure efficient conversion of Fe(OH)_3_ to Fe_2_O_3_ while maintaining good dispersion on the carrier surface. The optimal temperature facilitated the formation of well-dispersed Fe_2_O_3_ particles, which are crucial for high catalytic activity. On the other hand, both insufficient and excessive temperatures compromised the catalyst’s performance by either incomplete decomposition or particle agglomeration, respectively. Further analysis of the calcination time was conducted to study its impact on the catalytic performance, as shown in Figure 3F. It was observed that for calcination time up to 3 h, both iron loading and catalytic performance reached their maximum values. However, for a period exceeding this duration, a slight decrease in iron loading and a sharp decrease in catalytic performance were detected. This might be explained by the possibility that the matrix was slightly destroyed or Fe_2_O_3_ was converted into other forms under prolonged calcination times.

**Figure 4:**
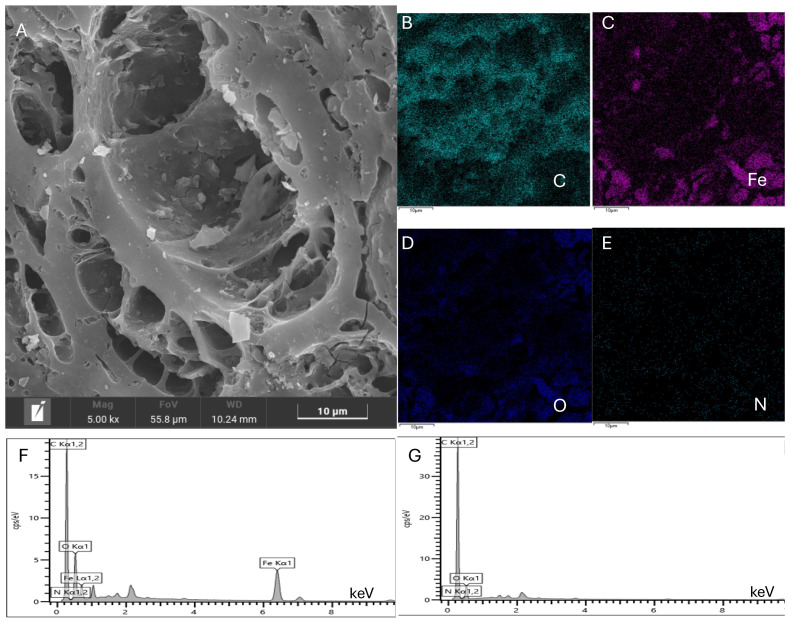
SEM analysis of Fe_2_O_3_/CAC. (**A**) Morphology analysis of Fe_2_O_3_/CAC; (**B**–**F**) Elemental analysis of Fe_2_O_3_/CAC; (**G**) Elemental analysis of CAC. The catalyst was prepared at optimal conditions: 0.6 mol/L of concentration of Fe(NO_3_)_3_, 0.6 mol/L of Na_2_CO_3_ (precipitant), and 350 °C for 3 h in the calcination process.

The SEM analysis was used to study the structure and morphology of Fe_2_O_3_/CAC, as shown in Figure 4. The pore channels were further enlarged, which might have been caused by the calcination process. The Fe_2_O_3_ particles were clearly observed, and most of them were evenly distributed inside the pores, maximizing the amount of effective active sites. The enlargement of the pore channels likely facilitated greater accessibility and dispersion of Fe_2_O_3_ particles within CAC. This enhanced distribution is crucial for catalytic efficiency as it increases the surface area available for reactions. The calcination process might have played a dual role by not only enlarging the pores but also helping to anchor the Fe_2_O_3_ particles firmly onto the CAC surface. Compared with the initial carrier, the elemental analysis of the catalyst also indicated that Fe_2_O_3_ was successfully immobilized on the surface of CAC, as shown in Figure 4B–G. The elemental mapping confirmed the uniform distribution of iron across the CAC surface, suggesting a successful synthesis process. This immobilization is essential for maintaining the stability and reusability of the catalyst, as it prevents the leaching of the active metal during catalytic cycles. These results provided strong evidence that the calcination process not only enhances the physical characteristics of the CAC but also effectively incorporates Fe_2_O_3_ particles, thereby creating a highly efficient catalyst.

### 2.3. Properties of Fe_2_O_3_/CAC

**Figure 5:**
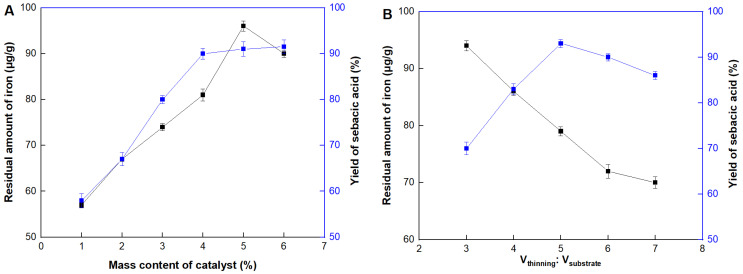

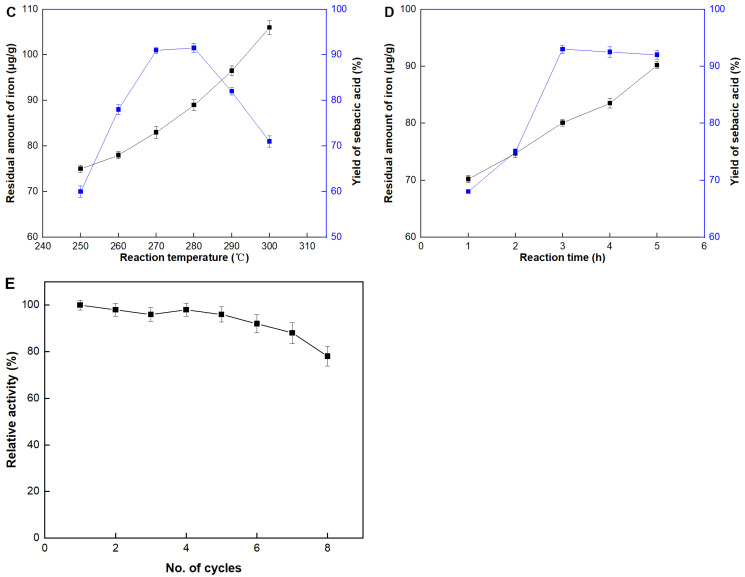
Properties of Fe_2_O_3_/CAC. Effect of (**A**) catalyst contents, (**B**) rations between the thinning agent and substrate castor oil, (**C**) reaction temperature, (**D**) reaction time, and (**E**) recycling number.

Although Fe_2_O_3_ was tightly attached on the carrier surface through the calcination, the slight leakage couldn’t be avoided during the reaction. Thus, the residual amount of iron in the product should be also detected to ensure the product’s safety. As shown in Figure 5A, the yield of sebacic acid reached the maximum value when the mass content of the catalyst was at 4%. After that, there was a slight change in the yield but would lead to a serious accumulation of iron in the product. The yield of sebacic acid reached 83.4%, which was increased 1.4 times than the traditional method (60.2%, as described in the Section 3.6 “Production of sebacic acid” using untreated Fe_3_O_4_). Moreover, the residual amount of iron was three times lower (by only about 80 μg/g), when Fe_2_O_3_/CAC was used, than that of the control experiment of Fe_2_O_3_ without immobilization (~240 μg/g). It might be explained that iron was evenly dispersed and tightly attached on the internal and out surface of the carrier, maximizing the effective catalytic sites and minimizing the possibility of leakage. Next, the reaction was carried out under different rations between the thinning agent and substrate castor oil (Figure 5B). The optimal ratio was 5. If it was above this value, although the residual amount of iron was also diluted, the concentrations of catalyst and substrate were also diluted, leading to the yield decrease.

The production of sebacic acid was evaluated at various temperatures, ranging from 250 to 300 °C, and the results were shown in Figure 5C. The optimum operational temperature was determined to be 270 °C. Increasing the temperature benefited the reaction by providing more energy to the reacting molecules. Essentially, higher temperatures led to faster molecular motion, resulting in more frequent and energetic collisions between reacting molecules, which in turn increased the rate of reaction. However, it was also observed that at temperatures higher than the optimum, there was a downside. The more energetic collisions, while beneficial for reaction rates, also increased the likelihood of iron leakage from the catalyst. This iron leakage was detected as a higher iron content in the product. The elevated temperatures likely caused the Fe_2_O_3_ particles to become less stable and more prone to detachment from the carbon-activated carrier (CAC), leading to contamination of the sebacic acid product with iron.

The reaction time was also evaluated, as shown in Figure 5D. The optimal operational time was determined to be 3 h, as extending the reaction time beyond this point did not result in any increase in the yield of sebacic acid. This reaction was conducted under high temperature and strong alkali conditions; thus, a longer reaction time would lead to iron leakage and potentially destroy the catalyst structure. The slight decrease in the yield of sebacic acid observed at 5 h might be attributed to unknown side reactions that could occur under prolonged exposure to these harsh conditions. Such side reactions could consume reactants or intermediates, thereby reducing the overall efficiency of sebacic acid production. This underscores the importance of carefully optimizing the reaction time to maximize yield while preserving catalyst integrity.

In industrial applications, recycling the catalyst is essential to reduce production costs. Consequently, the operational stability of the Fe_2_O_3_/CAC catalyst was examined, as shown in Figure 5E. The catalyst was collected after each batch, washed, and then added to fresh reactants. After five batches, a slight decrease in yield was observed, indicating an excellent operational stability of the catalyst. The slight decrease in yield over multiple batches could be attributed to iron leakage under the extreme reaction conditions. Despite this, the catalyst demonstrated a remarkable ability to retain its activity over repeated cycles, highlighting its potential for sustainable industrial application. This durability reduces the frequency of catalyst replacement, thereby lowering operational costs and enhancing the overall efficiency of the process.

## 3. Materials and Methods

### 3.1. Materials

Four kinds of commercially activated carbons (20–30 mesh) derived from coal, coconut shell, nutshell, and wood, were purchased from Gongyi Hengxin Co., Ltd. (Zhengzhou, China). Fe(OH)_3_, ethylene diamine tetraacetic acid (EDTA), castor oil (with 81.8% of ricinoleic acid) and other normal reagents with the analytical pure were purchased from China National Pharmaceutical Group Corporation.

### 3.2. Pretreatment of Activated Carbons

Ten grams of four kinds of commercially activated carbons derived from coal, coconut shell, nutshell and wood were respectively washed five times with distilled water. Then, they were put into 100 mL of distilled water and kept under boiling for 20 min. After cooling, they were further washed five times by distilled water and dried at 100 °C overnight.

For tests of acid washing, four washed activated carbons (6 g) were respectively dispersed in three kinds of 30 mL acid solution (10%) including nitric acid, hydrochloric acid, and sulfuric acid. The controlled trial was carried out in 30 mL of distilled water. During nitric acid washing, the acid concentration was investigated from 10% to 50% to wash the coal-derived activated carbon (CAC). Carriers were collected and washed until the pH of the eluent was close to neutral. All samples were dried at 100 °C overnight for further evaluation.

To test the ultrasonic treatment, 6 g of clear CAC were dispersed in 30 mL of distill water or nitric acid solution (30%) to be kept for 0–3 h. Then, carriers were collected and washed until it came to neutral. All samples were dried at 100 °C overnight for the further evaluation.

### 3.3. Analysis of Functional Groups in Activated Carbons

Buret titration was employed to analyze oxygen-containing functional groups of carriers [19]. One gram of sample was added into 25 mL of solutions (0.1 mol/L) of sodium hydroxide, sodium carbonate, sodium bicarbonate, and hydrochloric acid, respectively. The mixture was kept under 150 rpm at 25 °C for 24 h. The mixture was separated, and the supernatant was neutralized by 0.1 mol/L sodium hydroxide or hydrochloric acid solution.

### 3.4. Adsorption Ability of Activated Carbons for Fe^3+^

One gram of sample was added into 15 mL of the Fe(NO_3_)_3_ solution (0.2 mol/L). The mixture was kept under 150 rpm at 25 °C for 24 h. The mixture was separated, and the supernatant was analyzed by 0.1 mol/L EDTA solution [20].

### 3.5. Preparation of Catalyst

Six grams of clear CAC were dispersed in 30 mL of nitric acid solution (30%) to be kept for 1 h. CAC were washed until it came to neutral and dried at 100 °C overnight. Four grams of treated CAC were dispersed in 30 mL of Fe(NO_3_)_3_ solutions (0.1 to 1.0 mol/L) under 150 rpm at room temperature for 24 h. Five alkaline solutions (0.5 mol/L) were used as precipitants including sodium hydroxide, sodium carbonate, sodium bicarbonate, urea, and ammonia. All precipitant solutions was added drop by drop under 200 rpm until the precipitation does not increase. Then, the mixture was further kept for 24 h. After the centrifugation, precipitates were roasted under 350 °C for 3 h in the muffle furnace. The obtained catalyst was further washed with 0.1 mol/L NaOH solution and distilled water three times. Then, they were dried at 100 °C overnight.

### 3.6. Production of Sebacic Acid

Castor oil (30 mL) was added to 120 mL of NaOH solution (3 mol/L). The mixture was kept boiling for 40 min and cooled to 90 °C. Hydrochloric acid solution (6 mol/L) was employed to adjust pH to 6. The upper liquid was the ricinoleic acid solution. The prepared ricinoleic acid (15 mL) was added drop by drop into a mixture including 15 mL of 40% NaOH solution, 60 mL of liquid paraffin, and 0.75 g of our catalyst or traditional catalyst (untreated Fe_3_O_4_) under 280 °C at 200 rpm. After that, the mixture was further kept for 3 h. Then, the mixture was cooled to 85 °C and 300 mL of distill water (85 °C) was added. The whole mixture was boiled for 5 min. After that, the solution was centrifugated at 2000 rpm for 6 min. The solution was divided into three layers. The middle layer was collected. Hydrochloric acid solution (6 mol/L) was employed to adjust pH into 6. After filtering, hydrochloric acid solution (0.1 mol/L) was added until no increase in precipitates. Diethyl ether (2 × 5 mL) was used to extract the target product. The diethyl ether solution was dried by N_2_ and 5 mL of methanol was used to dissolve the product. The content of sebacic acid was analyzed by gas chromatography (GC) [14]. The yield of sebacic acid was calculated based on the initial content of effective substrate (ricinoleic acid) in castor oil. The convertion rate from ricinoleic acid to sebacic acid was 1:1 (molar ratio). Thus, the yield of sebacic acid was equal to the amount of sebacic acid divided by the initial amount of ricinoleic acid. The calculation unit was the molar.

### 3.7. GC-MS Analysis for Sebacic Acid

The method for GC analysis of castor oil was based on the previous work [10]. Sebacic acid was analyzed as follows: a Shimadzu 2030 GC and SH-Rtx-Wax (30 m × 0.25 mm × 0.25 µm, Shimadzu Co., Ltd., Kyoto, Japan) was used with nitrogen as a carrier gas (1.36 mL/min). The injection temperature was 250 °C. The oven temperature was held at 100 °C for 3 min and increased to 300 °C with a rate of 36 °C/min. The detector temperature was set as 300 °C. Detection was performed by means of a Shimadzu QP2020 NX single quadrupole detector (ionization energy, 70 eV; ion source, 250 °C; quadrupoles, 150 °C; transfer line, 280 °C; *m*/*z* 41–500). Peaks were identified with the help of NIST 20 s MS database.

### 3.8. Characterization Techniques

The morphology was investigated via a Carl Zeiss SIGMA (ZEISS, Jena, Germany) equipped with a field-emission gun operated at 5.0 kV. Thermogravimetric analysis (TGA) was carried out by STA 449F3 (Netzsch, Waldkraiburg, Germany).

## 4. Conclusions

This research successfully demonstrated an efficient and environmentally friendly method for the production of sebacic acid derived from castor oil using Fe_2_O_3_/CAC. Key to the process was the optimization of catalyst preparation and operational conditions. The chemical composition, structure, and morphology properties were investigated by different characterizations, such as scanning electron microscopy (SEM), and thermogravimetric analysis (TG/DTG). Sebacic acid was prepared with a satisfactory yield of 83.4% under the optimal condition, which was significantly increased than the traditional method. After being used five times, only a slight decrease was observed for the catalytic performances of Fe_2_O_3_. With excellent operational stability and reduced iron leakage, this catalyst system would be a viable solution for industrial scale-up. The study not only provided a promising candidate for the sebacic acid production but also contributed to the broader field of sustainable chemical manufacturing.

## Data Availability

The original contributions presented in the study are included in the article, further inquiries can be directed to the corresponding authors.
